# Cognitive Decline in Multiple Sclerosis Is Related to the Progression of Retinal Atrophy and Presence of Oligoclonal Bands: A 5-Year Follow-Up Study

**DOI:** 10.3389/fneur.2021.678735

**Published:** 2021-07-13

**Authors:** Natasa Giedraitiene, Egle Drukteiniene, Rasa Kizlaitiene, Andrius Cimbalas, Rimvydas Asoklis, Gintaras Kaubrys

**Affiliations:** ^1^Center of Neurology, Clinic of Neurology and Neurosurgery, Institute of Clinical Medicine, Faculty of Medicine, Vilnius University, Vilnius, Lithuania; ^2^Center of Eye Diseases, Clinic of Ear, Nose, Throat, and Eye Diseases, Institute of Clinical Medicine, Faculty of Medicine, Vilnius University, Vilnius, Lithuania

**Keywords:** multiple sclerosis, cognition, BICAMS, OCT, oligoclonal bands, brain atrophy

## Abstract

**Background:** Brain atrophy, which is associated with cognitive impairment and retinal nerve fiber layer (RNFL) atrophy, is the main biomarker of neurodegeneration in multiple sclerosis (MS). However, data on the relationship between inflammatory markers, such as oligoclonal bands (OCBs) in the cerebrospinal fluid (CSF), and cognition, RNFL atrophy, and brain atrophy are scarce. The aim of this study was to assess the influence of RNFL thickness, brain atrophy markers, intrathecal OCBs, and the immunoglobulin G (IgG) index on cognitive decline over a 5-year period in patients with MS.

**Methods:** This prospective, single-center, observational cohort study included 49 patients with relapsing MS followed up over 5 years. At baseline, the patients underwent brain magnetic resonance imaging (MRI). Cognitive evaluation was performed using the Brief International Cognitive Assessment for MS (BICAMS), and RNFL thickness was assessed using optical coherence tomography (OCT). OCBs and IgG levels in the CSF were evaluated at baseline. The BICAMS, OCT, and MRI findings were re-evaluated after 5 years.

**Results:** A significant reduction in information processing speed, visual learning, temporal RNFL thickness, the Huckman index, and third ventricle mean diameter was found in all 49 patients with relapsing MS over the observation period (*p* < 0.05). Of the patients, 63.3% had positive OCBs and 59.2% had elevated IgG indices. The atrophy of the temporal segment and papillomacular bundle and the presence of OCBs were significantly related to a decline in information processing speed in these patients (*p* < 0.05). However, brain atrophy markers were not found to be significant on the general linear models.

**Conclusions:** RNFL atrophy and the presence of OCBs were related to cognitive decline in patients with MS over a 5-year follow-up period, thereby suggesting their utility as potential biomarkers of cognitive decline in MS.

## Introduction

Multiple sclerosis (MS) is an inflammatory and neurodegenerative disease of the central nervous system (CNS) (1) that leads to demyelination and diffuse neurodegeneration in both the brain and spinal cord gray matter and white matter (1, 2). The course of the disease is usually relapsing-remitting from onset (1, 3). Studies have also shown the involvement of both inflammatory and neurodegenerative processes from the early stages of the disease (2, 4, 5). However, it remains unknown whether early degeneration is an independent process in MS or whether it is secondary to inflammation (2, 5). Inflammation in MS is more obvious and can be easily assessed, documented, and monitored in patients. In contrast, neurodegeneration is more difficult to assess and monitor, especially in the early stages of the disease (5).

Understanding the mechanism and causes of neurodegeneration in MS may be fundamental to developing therapies that can help halt this process and presumably prevent the progression of disability (2, 3). Brain atrophy assessed using magnetic resonance imaging (MRI) may be a biomarker for early neurodegeneration and may help predict the prognosis and disease course. Nevertheless, the measurement of atrophy on MRI in routine clinical practice remains a hurdle (6, 7). The identification of sensitive and accessible markers of and diagnostic tools for neurodegeneration may help us understand the relationship between these markers and may facilitate the development of easy-to-use and low-cost tools for exploring the pathophysiology of neurodegeneration in MS (2–4).

Cognitive impairment in MS reflects the underlying inflammatory and neurodegenerative pathological features of the disease (8). It is present in up to 50–70% of patients with MS and significantly lowers their quality of life (8, 9). The most frequently observed cognitive problems include deficits in information processing speed, episodic memory, complex attention, and executive function (8, 10, 11). The severity of cognitive impairment varies considerably among individuals and can be observed even in the early stages of the disease (12, 13). Brain imaging studies have demonstrated that cognitive impairment in MS is related to the loss of brain volume or brain atrophy, which is an important sign of neurodegeneration (8, 10). Cognitive impairment and brain atrophy have been classically considered as features that present in the advanced stages of the disease (14). However, numerous studies have demonstrated that both cognitive impairment and brain atrophy may occur in the early stages of the disease and even in clinically and radiologically isolated syndromes (15, 16).

Optical coherence tomography (OCT) measurements of the macular ganglion cell layer and retinal nerve fiber layer (RNFL) have been proposed as biomarkers of axonal damage in MS (17). Recently, retinal OCT has been used as a sensitive and practical alternative to MRI for the evaluation of neurodegeneration in MS (17, 18). However, studies have demonstrated a strong relationship between cognitive impairment across various cognitive domains and RNFL atrophy even in patients without MS-related optic neuritis (19–21). Some studies also indicate that RNFL thickness and cognition could be sensitive biomarkers that can be used for discriminating relapsing and progressive forms of the disease (21, 22). RNFL thickness may be associated with brain atrophy and cognitive impairment; therefore, OCT may be useful in assessing CNS neurodegeneration in MS (23, 24).

The presence of oligoclonal bands (OCBs) in the cerebrospinal fluid (CSF) or an elevated immunoglobulin G (IgG) index in patients with MS supports the diagnosis. Persistent intrathecal inflammation, demonstrated by the presence of OCBs in the CSF (25), is one of the hallmarks of MS in up to 95% of patients (25, 26). Previous research demonstrated that the presence of CSF-OCBs in patients with MS tends to be related to widespread cognitive changes, especially worse visual memory (27) and larger periventricular lesion area on MRI (28). However, data on the relationship between inflammatory markers, such as CSF-OCBs or elevated IgG indices, and neurodegenerative markers, such as brain atrophy markers or RNFL thickness, in MS are limited (27).

The aim of this study was to assess the impact of neurodegenerative markers, such as RNFL thickness and brain atrophy, as well as inflammatory markers, such as intrathecal OCBs and the IgG index, on the cognitive decline in patients with MS over a 5-year follow-up period.

## Materials and Methods

This prospective, single-center, observational cohort study was conducted at Vilnius University Hospital Santaros Klinikos, Lithuania. Patients were enrolled and assessed between 2012 and 2019. All patients signed an informed consent form, and the study was approved by the appropriate institutional review board. The inclusion criteria were as follows: age between 18 and 60 years, presence of relapsing-remitting MS, and absence of relapse and/or steroid treatment at least 30 days before the enrollment assessment and during the follow-up assessment. All patients were on stable-disease-modifying therapy at least 3 months before the assessment, and none had a history of MS-associated optic neuritis. The exclusion criteria were the presence of primary or secondary progressive MS, neurological disorders other than MS, any vision or hearing problems that could influence performance on the tests, and optic neuritis during the observation period.

After providing signed written informed consent, all the patients underwent physical and neurological examinations, neuropsychological assessment using the Brief International Cognitive Assessment for MS (BICAMS), ophthalmological examination using OCT, and brain MRI. The same evaluations were repeated 5 years (±14 days) later. The changes from baseline to the follow-up visit were calculated for all assessments.

All patients with MS were diagnosed according to the McDonald criteria by a neurologist at the Vilnius Multiple Sclerosis Center (29, 30). Neurological disability was assessed using the Expanded Disability Status Scale (EDSS) (31). The patients also underwent a lumbar puncture for evaluating the CSF-OCBs and IgG index at baseline.

### BICAMS

All the patients were examined by the same neurologist, and the tests were administered in the same sequence: the Symbol Digit Modalities Test (SDMT) to evaluate the information processing speed; the Brief Visuospatial Memory Test Revised (BVMT-R), i.e., the first three recall trials to evaluate visual learning and memory; and the California Verbal Learning Test, Second Edition (CVLT-II), i.e., the first five trials to evaluate verbal learning and memory (32–35). The baseline and follow-up assessments were performed by the same neurologist. Different versions of the BICAMS test were used during the baseline and follow-up assessments.

### OCT

OCT was performed on both eyes of each patient by using a spectral-domain OCT device (Spectralis, Heidelberg Engineering, Heidelberg, Germany), and the images were evaluated by the same ophthalmologist. RNFL thickness was measured using the RNFL-N axonal protocol with three 3.4-mm-diameter circular scans. The RNFL Spectralis protocol generates maps with four quadrants (superior, inferior, nasal, and temporal) and six sector thicknesses (superonasal, nasal, inferonasal, inferotemporal, temporal, and superotemporal); it also measures the thickness of the papillomacular bundle (PMB), the nasal-to-temporal ratio, and the average thickness.

### MRI

Brain MRI with gadolinium enhancement was performed in all patients by using a Siemens Aera 1.5 T MRI scanner (Siemens, Munich, Germany). MRI assessment included the following sequences: T1 (repetition time, 526 ms; echo time, 14 ms), T2 (repetition time, 4,110 ms; echo time, 105 ms), and fluid-attenuated inversion recovery (FLAIR) T2 (repetition time, 9,000 ms; echo time, 122 ms). A radiologist who was blinded to the patient's clinical data calculated the linear measures of brain atrophy. To evaluate brain atrophy, the Huckman index (sum of the greatest and smallest distances between the frontal horns), third ventricle width, and bicaudate ratio (BCR) were measured. The BCR was measured on a FLAIR axial image, where the heads of the caudate nuclei were best visible and closest to each other. The BCR was determined as the minimum intercaudate distance divided by the distance between the outermost parts of both the hemispheres measured along the same line.

### Statistical Analysis

Data were analyzed using IBM SPSS Statistics for Windows, Version 23.1 (IBM Corp., Armonk, NY, USA). Continuous variables were reported as medians and ranges or means and standard deviations, while categorical variables were reported as absolute numbers and percentages of total patients. The normal distribution of the data was verified using the Shapiro–Wilk test. Student's *t*-test was used to compare the means between the two groups (baseline and follow-up assessments). The chi-square test was used for categorical variables. General linear regression was used to assess the relationship between the change in cognitive functions over 5 years (dependent variable) and the following clinical and demographic factors as explanatory variables: the change in RNFL thickness over 5 years, the change in brain atrophy markers over 5 years, the presence of OCBs, the IgG index, disease duration, age, and sex. The dependent variables in the models were the changes in SDMT, BVMT-R, and CVLT-II over 5 years. The independent variables (regressors) were the changes in different segments of the RNFL over 5 years in both eyes; the changes in brain atrophy markers on brain MRI (third ventricle width, Huckman index, or bicaudal score) over 5 years; the difference in the EDSS score between the baseline and follow-up assessments; and the IgG index, the presence of OCBs, age, sex, and disease duration at baseline. A value of *p* < 0.05 was considered significant.

## Results

### Patients

**Figure 1:**
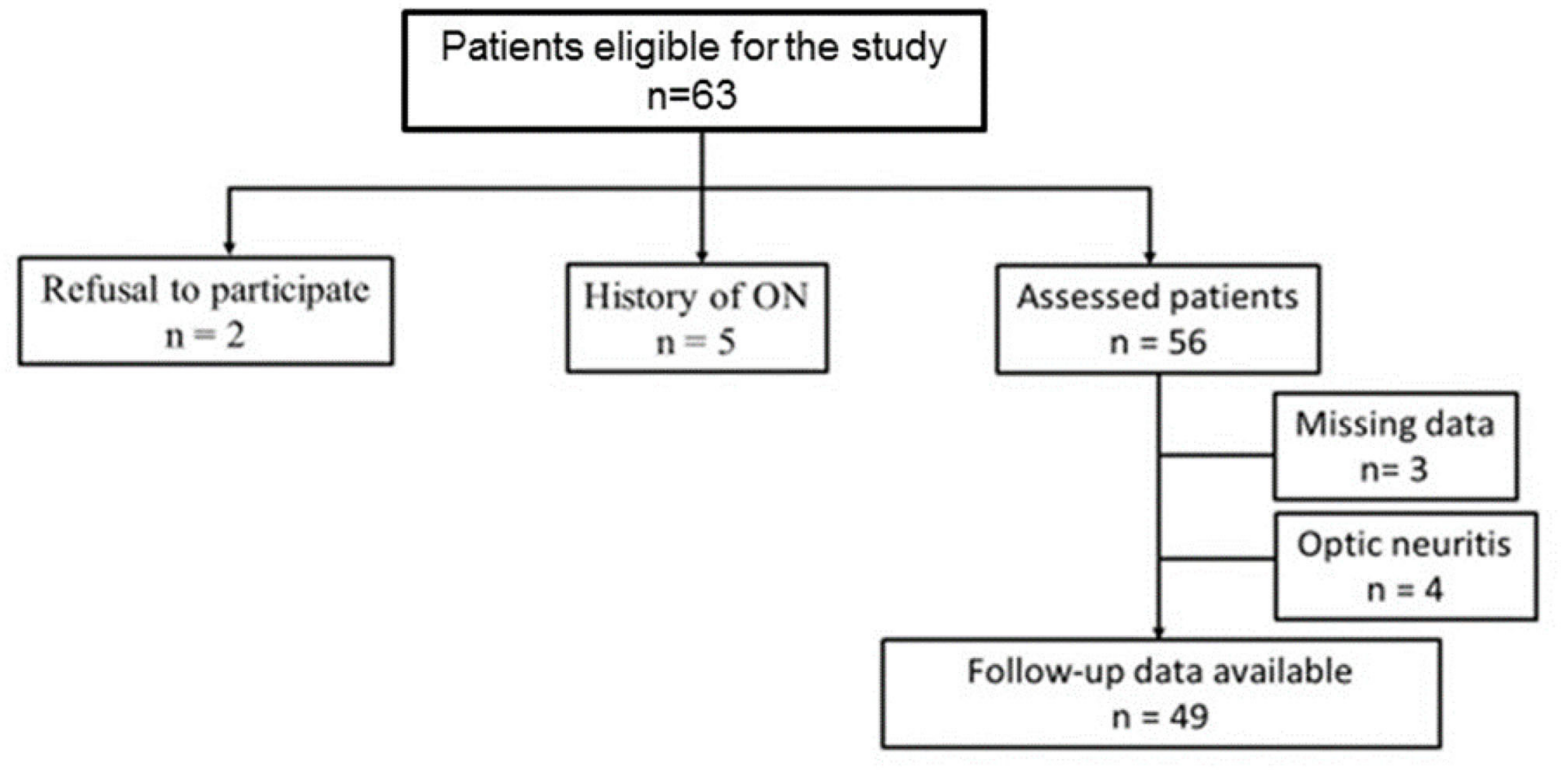
Flowchart illustrating patient selection. ON, optic neuritis.

Sixty-three patients were enrolled in this study. The 5-year follow-up data were available for 49 patients (77.8%) (Figure 1).

All patients had relapsing-remitting MS. The demographic and clinical characteristics of the patients are listed in Table 1.

**Table 1:** Clinical and demographic characteristics of the patients.

**Demographic and clinical variables**	***N***	**%**
Sex		
Female	37	75.5
Age (years)	47.3 ± 11.1	–
Disease duration (years)	11.4 ± 4.6	–
Education (years)	13.7 ± 3.2	–
EDSS		
Baseline assessment	2.8 ± 1.1	–
Follow-up assessment^*^	4.0 ± 1.4	–
Nonocular relapses^**^	2.2 ± 2.1	–
OCBs		
Positive	31	63.3
IgG index		
Elevated^***^	29	59.2

*EDSS, expanded disability status scale; OCBs, oligoclonal bands; IgG index, immunoglobulin G index*.

* *Follow-up was performed 5 years later*.

** *Relapses were assessed over 5 years (from baseline assessment up to the follow-up assessment)*.

*** *The IgG index was considered elevated when it was more than 0.77*.

### CSF Assessment

Of 49 patients, 63.3% had positive OCBs and 59.2% had elevated IgG indices in the CSF. Positive OCBs and elevated IgG indices did not differ according to sex (χ^2^ = 0.079, *p* > 0.05 and χ^2^ = 0.843, *p* > 0.05, respectively), age (*p* > 0.05), and disease duration (*p* > 0.05). The severity of disability was assessed using the EDSS at baseline, and the changes in EDSS scores between the baseline and follow-up assessments did not differ between patients with positive and negative OCBs (*p* > 0.05), as well as between patients with elevated and lower than normal IgG indices (*p* > 0.05). The incidence of positive OCBs and elevated IgG indices was similar in patients (63.3 and 59.2%, respectively); however, no relationship was found between positive OCBs and elevated IgG indices (χ^2^ = 0.993, *p* > 0.05).

### Cognitive Dynamics in Patients With MS

The scores of the SDMT and BVMT-R were significantly lower during the follow-up assessment than at the baseline assessment, while the CVLT-II scores did not differ between the baseline and follow-up assessments (Table 2).

**Table 2:** Cognitive scores at baseline and follow-up in patients with MS.

**Test**	**Baseline assessment**	Follow-up assessment^*^	p^**^
SDMT	44.5 ± 12.6	40.3 ± 12.6	** <0.001**
BVMT-R	24.7 ± 6.1	23.1 ± 7.2	** <0.05**
CVLT-II	59.5 ± 9.2	57.4 ± 11.5	>0.05

*SDMT, symbol digit modalities test; BVMT-R, brief visuospatial memory test revised; CVLT-II, california verbal learning test, Second Edition*.

* *Follow-up was performed 5 years after the baseline assessment*.

** *Student's t-test for paired samples. Bold values indicate significant differences or indicators*.

### RNFL Thickness Determined Using OCT

The average RNFL thickness in the temporal, nasal, inferotemporal, and inferonasal segments and the overall global average thickness were significantly lower in both eyes at the follow-up assessment (*p* < 0.05), while the average thickness of the PMB was lower in the right eye and the thickness of the superotemporal segment was lower in the left eye. The OCT results are presented in Table 3.

**Table 3:** Changes in RNFL thicknesses in patients with MS at the baseline and follow-up assessments.

**Segment**	**Right eye,** ΔRNFL_B-5_^*^ ± SD^**^	p^***^	**Left eye,** ΔRNFL_B-5_^*^ ± SD^**^	p^***^	**Both eyes,** ΔRNFL_B-5_^*^ ± SD^**^	p^***^
T	2.2 ± 4.1	** <0.001**	1.6 ± 4.9	** <0.05**	1.9 ± 3.7	** <0.001**
N	4.2 ± 4.9	** <0.001**	4.4 ± 5.5	** <0.001**	4.3 ± 4.3	** <0.001**
TS	1.1 ± 5.1	>0.05	1.8 ± 5.9	** <0.05**	1.4 ± 4.7	** <0.05**
TI	4.5 ± 6.5	** <0.001**	3.9 ± 7.4	** <0.001**	4.2 ± 5.4	** <0.001**
NS	−0.2 ± 5.0	>0.05	0.9 ± 6.0	>0.05	0.3 ± 4.2	>0.05
NI	4.3 ± 6.8	** <0.001**	4.0 ± 7.7	** <0.001**	4.2 ± 5.6	** <0.001**
PMB	1.9 ± 3.6	** <0.001**	0.7 ± 4.9	>0.05	1.3 ± 3.3	** <0.05**
G	2.8 ± 3.1	** <0.001**	2.7 ± 4.4	** <0.001**	2.8 ± 3.3	** <0.001**

*RNFL, retinal nerve fiber layer; SD, standard deviation; T, temporal; N, nasal; TS, superotemporal; TI, inferotemporal; NS, superonasal; NI, inferonasal; PMB, papillomacular bundle; G, global*.

* *Change from the baseline to follow-up assessments: the mean of delta*.

** *Standard deviation of delta*.

*** *Student's t-test for paired samples. Bold values indicate significant differences or indicators*.

### Linear Measures of Brain Atrophy

The Huckman index and third ventricle width were significantly lower during the follow-up assessment than at the baseline assessment. However, the BCR did not differ between the baseline and follow-up assessments (Table 4).

**Table 4:** Brain atrophy markers at the baseline and follow-up assessments in patients with MS.

**Brain atrophy marker**	**Baseline assessment**	Follow-up assessment^*^	p^**^
HI	49.3 ± 7.3	52.0 ± 8.3	** <0.001**
TVW	4.7 ± 1.9	6.3 ± 2.1	** <0.001**
BCR	0.1 ± 0.03	0.1 ± 0.04	>0.05

*HI, huckman index; TVW, third ventricle width; BCR, bicaudate ratio*.

* *Follow-up was performed 5 years after the baseline assessment*.

** *Student's t-test for paired samples. Bold values indicate significant differences or indicators*.

### Relationship of Disease Characteristics and Biomarkers of Neurodegeneration and Inflammation to Cognitive Decline

A general linear model was used to assess the relationship of the changes in RNFL thickness, brain atrophy markers, EDSS scores, OCBs, IgG index, and disease characteristics (age, sex, and disease duration) to the changes in cognitive domains over 5 years. The dependent variables in the models were the changes in SDMT, BVMT-R, and CVLT-II scores over 5 years. The independent variables (regressors) were the changes in different segments of the RNFL over 5 years, which were assessed as the changes in the mean values for both eyes; the changes in brain atrophy markers on brain MRI (third ventricle width, Huckman index, or bicaudal score) over 5 years; the differences in the EDSS scores between the baseline and follow-up assessments; and the IgG index, presence of OCBs, age, sex, and disease duration at baseline (Table 5). The decline in information processing speed over 5 years in patients with relapsing MS was explained by the RNFL thickness in the temporal segment or PMB in both eyes as well as the CSF-OCBs.

**Table 5:** Regression models that explain the cognitive decline over 5 years in patients with MS.

**Dependent variable**	**Regression model**	***R*2**	***p* (*R*^**2**^;** ** coefficients)**
Δ*SDMT*_B−5_	−3.1 − 1.0 × (ΔRNFL_T_B−5_) + 3.3 × CSF_OCBs	0.599	<0.01
Δ*SDMT*_B−5_	− 8.8 − 1.1 × (ΔRNFL_PMB_B−5_) + 4.4 × CSF_OCBs	0.480	<0.01

*R2, coefficient of determination; ΔSDMT_B−5_, difference in SDMT results between the baseline and follow-up assessments; SDMT, symbol digit modalities test; ΔRNFL_T_B−5_, the difference in temporal segment thickness assessed as the change in the mean of the value for both eyes between the baseline and follow-up assessments; ΔRNFL_PMB_B−5_, the difference in papillomacular bundle thickness assessed as the change in the mean of the value for both eyes between the baseline and follow-up assessments; CSF_OCBs, oligoclonal bands in the cerebrospinal fluid*.

## Discussion

Cognitive impairment, RNFL thickness, and brain atrophy are markers of neurodegeneration in MS (21, 29, 36, 37), whereas positive OCBs and elevated IgG indices in the CSF are markers of inflammation (25, 26). MRI was long considered the gold standard for monitoring the degenerative component of MS (6, 7). Thereafter, RNFL thickness and cognition were recognized as biomarkers of neurodegeneration (14, 17, 18). The presence of CSF-OCBs in patients with MS is supportive of the diagnosis (29, 30), even though the relationship between the patient's clinical and cognitive features has not been thoroughly examined. In our study, positive OCBs were detected in 63.3% of patients and elevated IgG indices were detected in 59.2%. CSF biomarkers such as OCBs and elevated IgG indices were not correlated with each other. Nevertheless, both are markers of inflammation and both are supportive of a diagnosis of MS (30). Previously published data regarding the correlation between the presence of OCBs and elevated IgG levels differ among studies; while some studies have reported positive correlations (38), others have not found any relationship (39–41). Moreover, in most patients with MS, when the number of OCBs is >2, no linear association is observed between CSF IgG levels and the number of OCBs (39, 40). The absence of such a correlation is possible because OCBs reflect the production of several monoclones, while the IgG index is a general indicator of enhanced autoimmune response. In our study, we did not find a correlation between the presence of OCBs and the IgG index.

We investigated whether cognitive decline over 5 years in patients with relapsing MS can be explained using neurodegenerative and inflammatory markers such as OCBs and IgG indices in the CSF. In the recently published revision of the McDonald diagnostic criteria, the detection of oligoclonal IgG bands in the CSF has regained importance (30). Therefore, we decided to assess the impact of inflammatory markers on cognitive decline. We found that among the biomarkers of neurodegeneration and neuroinflammation, RNFL thickness in the temporal segment, PMB thickness in both eyes, and the presence of OCBs were explanatory variables indicating a decline in information processing speed in patients with MS.

Many studies have provided data on one particular cognitive measure, i.e., the SDMT, which is considered particularly sensitive to the decrease in information processing speed that is commonly seen in MS (42, 43). Owing to its high reliability, validity, sensitivity, and specificity, the SDMT has demonstrated superiority over other cognitive tests for MS in recent years (43). Our findings are consistent with previously published data (42–44), and the SDMT was the only cognitive test in which the results were related to other markers of neurodegeneration and inflammation in our cohort. Our data also confirmed the association between cognitive function and RNFL thickness. In particular, we found that the average thickness in the temporal segment and PMB in both eyes was the most important OCT measure related to cognitive decline in our patients. During the past decade, OCT has developed into a sensitive method for imaging neurodegeneration in MS (17, 45). Studies have demonstrated that lower average temporal RFNL thickness correlates with a more active disease course, higher EDSS at the time of assessment, and greater EDSS score increase over time (37, 46). Correlations were also found between RNFL thickness and performance on some tests of cognitive function in patients with MS, particularly the SDMT (46, 47). Our results are in line with these previously published data showing that the SDMT score and RNFL thickness in the temporal segment are significant cognitive and ophthalmological indicators of neurodegeneration in MS (46, 47).

The limitations of our study were the relatively small sample size and the lack of a control group. However, we did not identify any controlled study in which a comparison group was used to assess the presence of OCBs and IgG indices in the CSF or in which the patients were followed up for a long duration of 5 years.

Another innovative aspect of our work was the combined analysis of inflammatory OCBs, neurodegeneration-related RNFL thickness, and cognition. The dependence of cognition on the presence of OCBs and RNFL thickness has not been previously investigated. We found a relationship between both neurodegenerative and inflammatory markers and information processing speed. RNFL thickness in the temporal segment, PMB thickness, and the presence of OCBs could be considered biomarkers in the diagnostic workup for MS. We did not detect a significant influence of any other RNFL segment thickness or brain linear measurement on cognition in our cohort of patients with MS. Our results confirm that the BICAMS and OCT measure different aspects of neurodegeneration and that the thinning of the RNFL is a potential biomarker for cognitive disability in MS (23, 47), because we found that cognitive decline may be predicted not only by markers of degeneration but also by markers of intrathecal inflammation. These results imply that both the thinning of the RNFL and the presence of CSF-OCBs are feasible biomarkers for cognitive decline in MS.

## Data Availability Statement

The original contributions presented in the study are included in the article/Supplementary Material, further inquiries can be directed to the corresponding author.

## Ethics Statement

The studies involving human participants were reviewed and approved by The Lithuanian Bioethics Committee approved the study in 2011 (2011-01-27 No.: L-12-01/2), the permission to continue the study was granted by the Lithuanian Bioethics Committee in 2018 (2018-02-22 No.: 6B-18-41). The patients/participants provided their written informed consent to participate in this study.

## Author Contributions

NG contributed to the conception and design of the study, acquisition of the data, analysis and interpretation of the data, and drafting of the manuscript. ED contributed to the analysis and interpretation of the data and to the conception and design of the study. RK contributed to the conception and design of the study and the revision of the manuscript. AC contributed to the analysis and interpretation of the data and drafting of the manuscript. RA contributed to the analysis and interpretation of the data and drafting of the manuscript. GK contributed to the conception and design of the study, analysis and interpretation of the data, and drafting of the manuscript. All authors discussed the results and contributed to and approved the final manuscript.

## Conflict of Interest

The authors declare that the research was conducted in the absence of any commercial or financial relationships that could be construed as a potential conflict of interest.

## Abbreviations

OCB: oligoclonal band
BICAMS: Brief International Cognitive Assessment for Multiple Sclerosis
EDSS: Expanded Disability Status Scale
SDMT: Symbol Digit Modalities Test
BVMT-R: Brief Visuospatial Memory Test Revised
CVLT-II, California Verbal Learning Test: Second Edition
PMB: Papillomacular Bundle
BCR: Bicaudate Ratio.
